# Automatic contouring QA method using a deep learning–based autocontouring system

**DOI:** 10.1002/acm2.13647

**Published:** 2022-05-17

**Authors:** Dong Joo Rhee, Chidinma P. Anakwenze Akinfenwa, Bastien Rigaud, Anuja Jhingran, Carlos E. Cardenas, Lifei Zhang, Surendra Prajapati, Stephen F. Kry, Kristy K. Brock, Beth M. Beadle, William Shaw, Frederika O'Reilly, Jeannette Parkes, Hester Burger, Nazia Fakie, Chris Trauernicht, Hannah Simonds, Laurence E. Court

**Affiliations:** ^1^ The University of Texas Graduate School of Biomedical Sciences at Houston Houston Texas USA; ^2^ Department of Radiation Physics Division of Radiation Oncology The University of Texas MD Anderson Cancer Center Houston Texas USA; ^3^ Department of Radiation Oncology The University of Texas MD Anderson Cancer Center Houston Texas USA; ^4^ Department of Imaging Physics The University of Texas MD Anderson Cancer Center Houston Texas USA; ^5^ Department of Radiation Oncology Stanford University School of Medicine Stanford California USA; ^6^ Department of Medical Physics (G68) University of the Free State Bloemfontein South Africa; ^7^ Division of Radiation Oncology and Medical Physics University of Cape Town and Groote Schuur Hospital Cape Town South Africa; ^8^ Division of Medical Physics Stellenbosch University Tygerberg Academic Hospital Cape Town South Africa; ^9^ Division of Radiation Oncology Stellenbosch University Tygerberg Academic Hospital Cape Town South Africa

**Keywords:** auto‐contour, deep learning, similarity metrics

## Abstract

**Purpose:**

To determine the most accurate similarity metric when using an independent system to verify automatically generated contours.

**Methods:**

A reference autocontouring system (primary system to create clinical contours) and a verification autocontouring system (secondary system to test the primary contours) were used to generate a pair of 6 female pelvic structures (UteroCervix [uterus + cervix], CTVn [nodal clinical target volume (CTV)], PAN [para‐aortic lymph nodes], bladder, rectum, and kidneys) on 49 CT scans from our institution and 38 from other institutions. Additionally, clinically acceptable and unacceptable contours were manually generated using the 49 internal CT scans. Eleven similarity metrics (volumetric Dice similarity coefficient (DSC), Hausdorff distance, 95% Hausdorff distance, mean surface distance, and surface DSC with tolerances from 1 to 10 mm) were calculated between the reference and the verification autocontours, and between the manually generated and the verification autocontours. A support vector machine (SVM) was used to determine the threshold that separates clinically acceptable and unacceptable contours for each structure. The 11 metrics were investigated individually and in certain combinations. Linear, radial basis function, sigmoid, and polynomial kernels were tested using the combinations of metrics as inputs for the SVM.

**Results:**

The highest contouring error detection accuracies were 0.91 for the UteroCervix, 0.90 for the CTVn, 0.89 for the PAN, 0.92 for the bladder, 0.95 for the rectum, and 0.97 for the kidneys and were achieved using surface DSCs with a thickness of 1, 2, or 3 mm. The linear kernel was the most accurate and consistent when a combination of metrics was used as an input for the SVM. However, the best model accuracy from the combinations of metrics was not better than the best model accuracy from a surface DSC as an input.

**Conclusions:**

We distinguished clinically acceptable contours from clinically unacceptable contours with an accuracy higher than 0.9 for the targets and critical structures in patients with cervical cancer; the most accurate similarity metric was surface DSC with a thickness of 1, 2, or 3 mm.

## 1 INTRODUCTION

Autocontouring systems using deep learning algorithms are widely available and have achieved great success, but it is almost impossible to develop a flawless autocontouring system. The AAPM Task Group 275 recently reported that failures in detecting contouring errors in treatment targets and normal structures are the largest and seventh largest risk factors in radiotherapy planning, respectively,^1^ and this risk will remain the same for autocontouring systems without an automatic contouring error detection system. To reduce the risk from contouring errors, automatic contouring error detection methods have been studied by several research groups. For example, Chen et al.^2^ extracted the geometric features of organs and developed machine learning–based contouring error detection models for normal structures in the head and neck. McIntosh et al.^3^ used image features to train a conditional random forest algorithm to detect contouring errors in thoracic structures. Hui et al.^4^ used contour shapes in principal component and Procrustes analysis to detect contouring errors in pelvic structures. We previously demonstrated^5^ that calculating the Dice similarity coefficient (DSC) between two independently generated contours can be used to detect errors in one of the contours.

In this work, we extended our previous study; we have calculated multiple quantitative metrics, instead of just utilizing DSC, to measure the similarity between two independently generated contours and used these metrics to provide quality assurance (QA) for the target and normal structure contours necessary for radiotherapy planning for cervical cancer. Cervical cancer was specifically chosen to test our QA method, because it was one of the first few sites where we have more than one autocontouring system independently developed in our clinic.

In this study, we hypothesized that if a contour is clinically unacceptable (i.e., the contour cannot be used clinically based on an experienced radiation oncologist's judgment), the discrepancy between the clinically unacceptable and a clinically acceptable contour will be substantial. This discrepancy can be quantified using the similarity metrics of the two contours, and errors in contours can be automatically reported by analyzing these metrics. With this method, we could automatically detect errors in an autocontour using another autocontour from an independent system. Even if both autocontours fail simultaneously, it is very unlikely that they will fail similarly based on our previous study with head‐and‐neck normal structures^5^; thus, the discrepancy between the two contours will still be substantial.

This study examined how to optimally flag incorrect contours by evaluating 11 different comparison metrics and evaluating different approaches to combining these metrics. The advantage of this method is that we could utilize any two independently developed autocontouring systems to perform contour QA. Commercial autocontouring systems are prevalent and many clinics have their own in‐house autocontouring systems nowadays. On the other hand, developing a classification algorithm for clinically acceptable and unacceptable contours is challenging as it requires numerous clinically unacceptable contours, which are clinically rare. Moreover, our QA method will be more useful with more advanced autocontouring systems, as less frequent failures in secondary autocontours would reduce false positives (clinically acceptable reference contours classified as clinically unacceptable due to failure in secondary autocontours); the performance of autocontouring systems is improving rapidly with advanced deep learning architectures and accumulated clinical data overtime.^6^

## 2 METHODS

To evaluate our QA method, we tested 11 quantitative metrics on 6 structures in the female pelvis: UteroCervix (uterus + cervix), CTVn (nodal clinical target volume [CTV]), PAN (para‐aortic lymph nodes), bladder, rectum, and kidneys, which are the fundamental normal structures to create intensity‐modulated radiation therapy or volumetric modulated arc therapy radiation treatment plans for cervical cancer. The femurs were excluded as our autocontouring systems barely failed for these structures unless there are imaging artifacts caused by high‐density materials (e.g., hip implants). As the imaging artifacts can be separately managed by an independent artifact detection algorithm,^7^ we did not include the femurs in this study.

### 2.1 Two deep learning–based autocontouring systems

The reference autocontouring system, which was used to generate the contours for clinical use, was developed in our previous study.^8^ The verification autocontouring system, which was used to test the clinical acceptability of the contours from the reference system, was developed by Rigaud et al.^9^ The two autocontouring systems were developed using the two independent training datasets, and the training datasets were created using the same contouring guideline.

As the nodal CTV and the PAN were not available in the original verification system, we trained the autocontouring models for the 2 structures using 140 CT scans to match all the structures. We used V‐Net^10^ and FCN‐8s^11^ architectures to train the nodal CTV and the PAN, respectively, to optimize the performance of the autocontouring system as described in our previous study.^8^

### 2.2 Data acquisition for the machine learning model

To train an algorithm to distinguish between clinically acceptable and unacceptable contours, we needed reference and verification contours in the same patients and organs. We created both reference and verification autocontours on 49 CT scans from MD Anderson (internal data) and 38 CT scans from 3 hospitals in South Africa (external data). Then, the reference autocontours, the ones that will be clinically used, were evaluated to determine whether they are clinically acceptable. For these 49 internal and 38 external CT scans, the quality of each reference autocontour was scored by one experienced radiation oncologist and one radiation oncology resident at MD Anderson. They each reviewed a subset of the contours and scored the contours as either needing no edits, minor edits, or major edits. For the contours scored as needing minor edits, revisions were preferred but not mandatory for the contours to be clinically acceptable, so the contours scored as needing major edits were considered clinically unacceptable contours.

**FIGURE 1:**
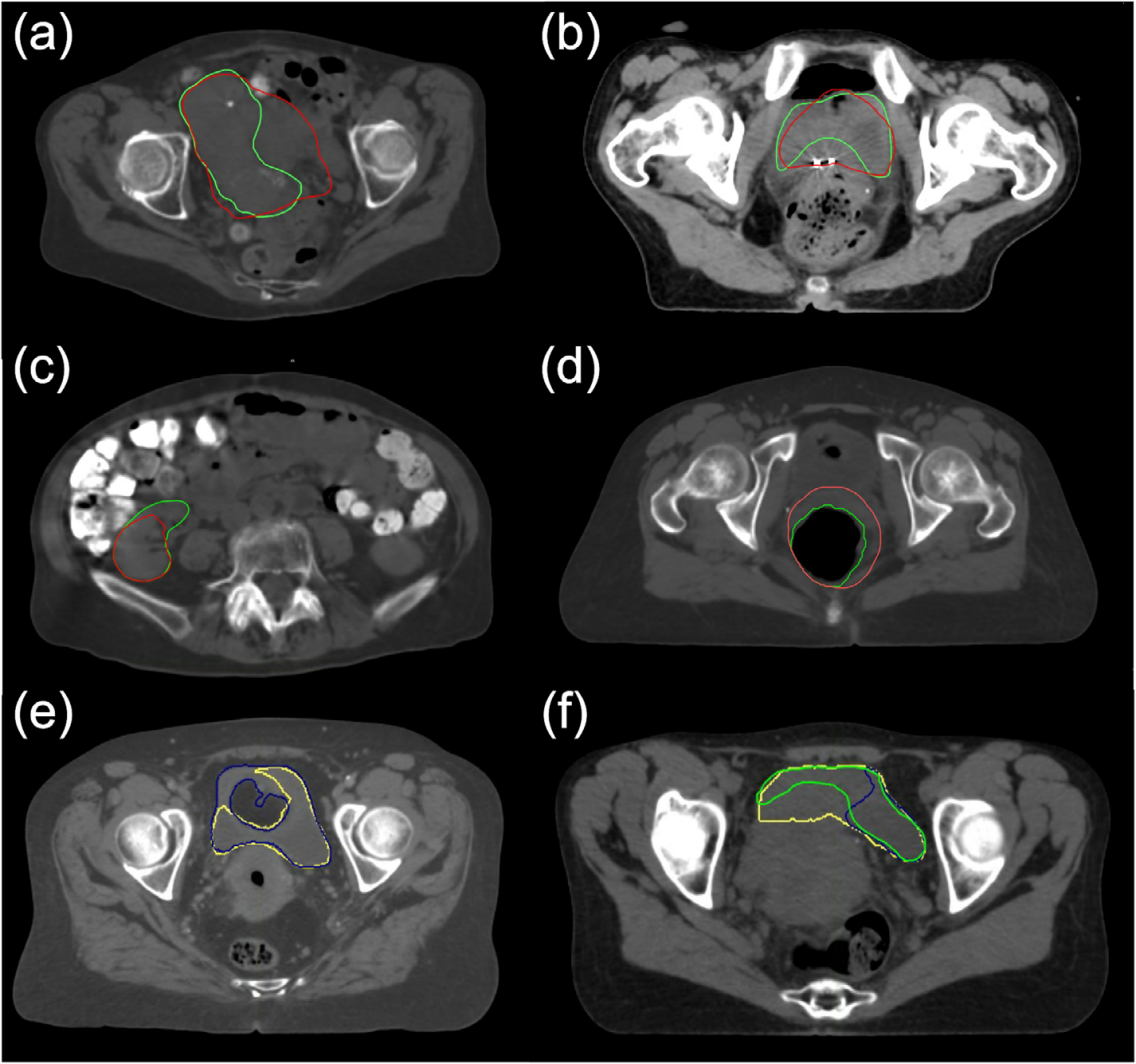
Examples of manually generated, clinically acceptable (green) and unacceptable (red) contours for the (a) UteroCervix, (b) bladder, (c) right kidney, and (d) rectum. (e) The reference autocontour (yellow) was clinically unacceptable when the verification autocontour (blue) was clinically acceptable. (f) Both the reference and the verification autocontours were clinically unacceptable

Furthermore, clinically acceptable and unacceptable contours for the 49 internal CT scans were manually created as reference contours by radiation oncology residents at MD Anderson. The clinically unacceptable contours were manually introduced to mimic a potential error that can be made by a human or a deep learning algorithm as a result of a lack of experience or an unclear soft tissue border, as illustrated in Figure 1. As most of the reference autocontours from the internal and external CT scans were clinically acceptable, the number of clinically unacceptable contours was not sufficient to determine the robust thresholds. These manually generated contours were added to the dataset to fill this gap and, therefore, enable the model to distinguish clinically acceptable and unacceptable contours more robustly.

**FIGURE 2:**
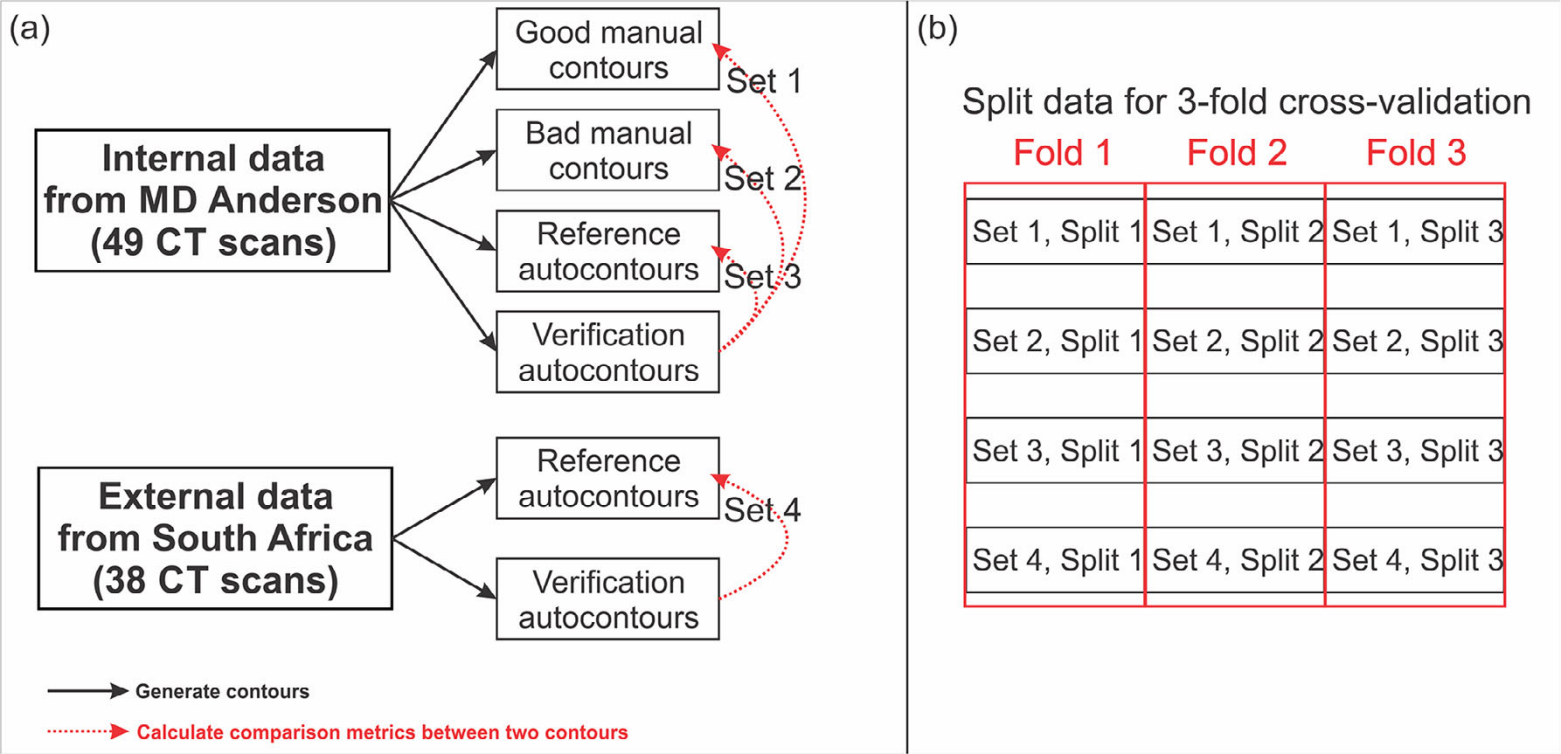
(a) Diagram demonstrating the data acquisition process for automatic contour QA model development and (b) demonstrating that each set was split equally into three for threefold cross‐validation. QA, quality assurance

Then, the quantitative metrics were calculated between the verification and the reference autocontours for the internal and external dataset, between the verification autocontours and the clinically acceptable manual contours for the internal dataset, and between the verification autocontours and the clinically unacceptable manual contours for the internal dataset, as shown in Figure 2a. In total, this resulted in 185 calculated data points per metric per structure from the 4 sets of data. Each set of data was split equally into three for threefold cross‐validation, as shown in Figure 2b. The results presented in this paper are the average of the threefold cross‐validation results.

We chose the 49 internal CT scans from a subset of the training dataset of the verification autocontouring system. As a result, all the verification contours for those 49 internal CT scans were clinically acceptable. This provided the distributions of the similarity between clinically acceptable verification contours and clinically acceptable manual contours, clinically unacceptable manual contours, and the reference autocontours.

### 2.3 Quantitative metrics

To quantify the similarities between the paired contours, we used four widely used conventional metrics for contour comparison studies: DSC,^12^ Hausdorff distance^13^ (HD_100), 95% Hausdorff distance (HD_95), and mean surface distance^14^ (MSD). We also tested the surface DSC, as suggested by Nikolov et al.^15^ (code available at https://github.com/deepmind/surface‐distance), with 1‐, 2‐, 3‐, 4‐, 5‐, 7‐, and 10‐mm shell thicknesses. All of the metrics were calculated for the 3D contours; therefore, we obtained one metric per patient per structure.

The definition of surface DSC with the tolerance *τ* is

$$Surface\_DSC\left(X,Y,\tau \right)=\frac{\left|X\cap Y_{B}^{\tau }\right|+\left|Y\cap X_{B}^{\tau }\right|}{\left|X\right|+\left|Y\right|}$$

where *A* and *B* are the volumes defined by the reference and the verification contours, *X* and *Y* are the surfaces of the volumes *A* and *B* , respectively, and $X_{B}^{\tau }$ includes the border region of the surface *X* and the border region consists of all of the points that are within the tolerance distance *τ* from the surface *X*. Based on the definition, if the tolerance is too high (e.g., 50 mm) or too low (e.g., 0.1 mm), the surface DSC value will be insensitive to change in contours at all or too sensitive to a subpixel level change, respectively. Therefore, the tolerances within a reasonable range were tested. The acronym for the surface DSC with *n*‐mm tolerance is represented as SDSC_*n* in this paper.

### 2.4 Error detection model with support vector machine

We used a support vector machine (SVM),^16^, ^17^ a machine learning classification algorithm, to determine the optimized hyperplane that distinguishes clinically acceptable and unacceptable contours from the training dataset. We then applied this SVM model to the validation dataset to calculate the SVM model (i.e., hyperplane) performance. We chose the SVM classification algorithm because it was the best performing machine learning algorithm for this task in our preliminary study and is computationally fast. Furthermore, SVM is one of the most intuitive classification algorithms,^18^ making it easy to interpret the derived hyperplane.

We tested various combinations of metrics to find the best metrics for the contouring error detection SVM model. We first tested the SVM models derived from the 11 quantitative metrics individually (single‐metric analysis) with the linear kernel, as this is the only kernel possible for a 1D input. We tested the values of the penalty parameter *C* from 1 to 50 and applied the best value to calculate the final accuracies. To provide a more comprehensive evaluation of the performance, we also performed an ROC analysis and calculated the area under the ROC curve (AUC) on each metric and each structure.

We also tested combinations of the 11 quantitative metrics on the basis of the results of the single‐metric analysis (multi‐metric analysis). The combinations tested are presented in Table 1. We tested linear, polynomial (with degree = 3), radial basis function, and sigmoid kernels in a multi‐metric analysis.

**TABLE 1:** List of the combined metrics used in the multi‐metric analysis

Name	Metrics used	Description
DSC_HD	DSC, HD_100	Most used quantitative metrics
Three_SDSC	SDSC 1, 2, 3 mm	Top three SDSC from single‐metric analysis
Five_SDSC	SDSC 1, 2, 3,4, 5 mm	Top five SDSC from single‐metric analysis
Four_metrics	DSC, HD_100, HD_95, MSD	Four conventional quantitative metrics
Five_metrics	DSC, MSD, SDSC 1, 2, 3 mm	Two most effective conventional metrics + three most effective SDSCs
Seven_metrics	DSC, MSD, SDSC 1, 2, 3, 4, 5 mm	Two most effective conventional metrics + five most effective SDSCs
Nine_metrics	DSC, MSD, SDSC 1, 2, 3, 4, 5, 7, 10 mm	Two most effective conventional metrics + all SDSCs
All_metrics	DSC, HD_100, HD_95, MSD, SDSC 1, 2, 3, 4, 5, 7, 10 mm	All available metrics

Abbreviations: DSC, Dice similarity coefficient; HD, Hausdorff distance; MSD, mean surface distance; SDSC, surface Dice similarity coefficient.

## 3 RESULTS

### 3.1 Single‐metric analysis

**FIGURE 3:**
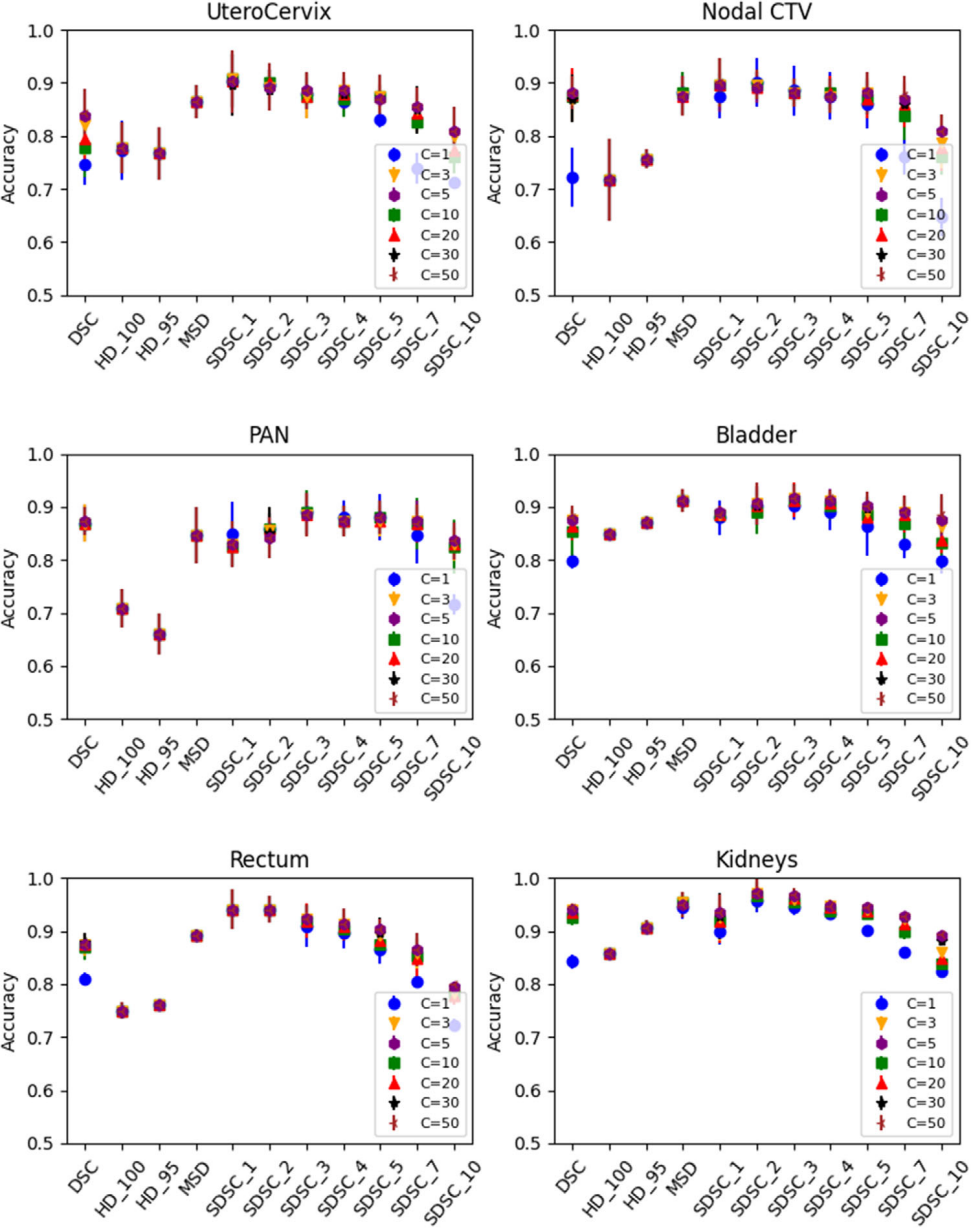
Average accuracies of the contour QA model with an individual metric for each structure with various penalty parameters, *C*. The error bar represents ±1 standard deviation from threefold cross‐validation. QA, quality assurance

The average accuracy (probability of differentiating the contour status correctly) of the threefold cross‐validation results is shown in Figure 3 for the 11 quantitative metrics that were tested individually with an SVM algorithm using a linear kernel with various penalty parameters, *C*, from 1 to 50. Overall, SDSC_1, SDSC_2, and SDSC_3 were the most accurate indicators in detecting contouring errors. The penalty parameters *C* = 10 gave the best results for these 3 metrics on average, although there were no substantial differences between the penalty parameters in between 3 and 50. Therefore, we presented all the accuracies with the penalty parameter *C* = 10.

The highest accuracy result was higher than 0.9 for the UteroCervix (0.91 ± 0.05 with SDSC_1), the nodal CTV (0.90 ± 0.03 with SDSC_1 and SDSC_2), the bladder (0.92 ± 0.03 with SDSC_3), the rectum (0.94 ± 0.04 with SDSC_1), and the kidneys (0.97 ± 0.03 with SDSC_2) and almost 0.9 for the PAN (0.89 ± 0.04 with SDSC_3).

The accuracy decreased as the tolerance for the surface DSC increased after 3 mm. DSC and MSD also accurately predicted the clinical acceptability of the contours. On the other hand, HD_100 and HD_95 were not as accurate as the other metrics.

To investigate the stability of each metric in response to small changes in the threshold, we calculated the average of the thresholds for four major metrics (DSC, SDSC_1, SDSC_2, and SDSC_3) over the structures and calculated the accuracy using the average thresholds on each structure (0.75, 0.30, 0.54, and 0.69 for DSC, SDSC_1, SDSC_2, and SDSC_3, respectively). The changes in the thresholds could exceed 20%, but the overall accuracy barely changed, as shown in Table 2. The accuracy was only reduced by a couple of percent in most cases. In the worst case, the accuracy was reduced by 8% when the threshold changed by 21.7% for the PAN. Although the change in thresholds might have decreased the specificity (probability of a clinically acceptable contour given that the contour is acceptable) and increased the sensitivity (probability of a clinically unacceptable contour given that the contour is unacceptable) or vice versa, the overall accuracy did not fluctuate according to small changes in the threshold. Furthermore, the accuracies, sensitivities, and specificities with SDSC_2 were calculated in three different scenarios: Accuracy was maximized with the SVM, sensitivity was fixed to be 0.90, and sensitivity was fixed to be 0.95, as shown in Table 3. Compared to the maximized accuracy, the accuracy was dropped by 6.5% and 12.0% on average when sensitivity was fixed to be 0.90 and 0.95, respectively.

**TABLE 2:** Changes in accuracy when applying the average threshold of various structures instead of optimal thresholds for each structure

Change in accuracy (∆Threshold) (%)	UteroCervix	CTVn	PAN	Bladder	Rectum	Kidneys
DSC	0.82 → 0.79 (4.5%)	0.87 → 0.88 (1.5%)	0.87 → 0.76 (17.0%)	0.88 → 0.86 (3.7%)	0.88 → 0.88 (9.8%)	0.94 → 0.87 (11.4%)
SDSC_1	0.91 → 0.91 (9.5%)	0.90 → 0.91 (2.0%)	0.83 → 0.81 (18.3%)	0.89 → 0.90 (7.5%)	0.94 → 0.93 (8.8%)	0.93 → 0.92 (2.9%)
SDSC_2	0.89 → 0.89 (0.2%)	0.90 → 0.89 (0.2%)	0.86 → 0.79 (20.2%)	0.91 → 0.90 (0.4%)	0.94 → 0.93 (10.8%)	0.97 → 0.93 (20.5%)
SDSC_3	0.88 → 0.88 (0.0%)	0.74 → 0.74 (1.2%)	0.88 → 0.80 (21.7%)	0.92 → 0.90 (8.1%)	0.92 → 0.92 (8.9%)	0.96 → 0.93 (15.4%)

Abbreviations: CTVn, nodal CTV; DSC, Dice similarity coefficient; PAN, para‐aortic lymph nodes; SDSC, surface Dice similarity coefficient.

**TABLE 3:** Overall accuracies, sensitivities, and specificities with maximized accuracy through the SVM, fixed sensitivity of 0.90, and fixed sensitivity of 0.95 when surface DSC with a thickness of 2 mm was used

SDSC_2	Maximize accuracy			Sensitivity ≥0.90			Sensitivity ≥0.95		
	Accuracy	Sensitivity	Specificity	Accuracy	Sensitivity	Specificity	Accuracy	Sensitivity	Specificity
UteroCervix	0.89	0.79	0.94	0.90	0.90	0.90	0.86	0.95	0.81
CTVn	0.90	0.78	0.97	0.80	0.91	0.74	0.72	0.96	0.59
PAN	0.86	0.68	0.95	0.67	0.90	0.56	0.62	0.95	0.46
Bladder	0.91	0.79	0.97	0.85	0.90	0.83	0.79	0.95	0.72
Rectum	0.94	0.86	0.97	0.89	0.90	0.88	0.79	0.96	0.72
Kidney	0.97	0.90	0.99	0.97	0.90	0.99	0.97	0.95	0.97

Abbreviations: CTVn, nodal CTV; DSC, Dice similarity coefficient; PAN, para‐aortic lymph nodes; SDSC, surface Dice similarity coefficient; SVM, support vector machine.

**FIGURE 4:**
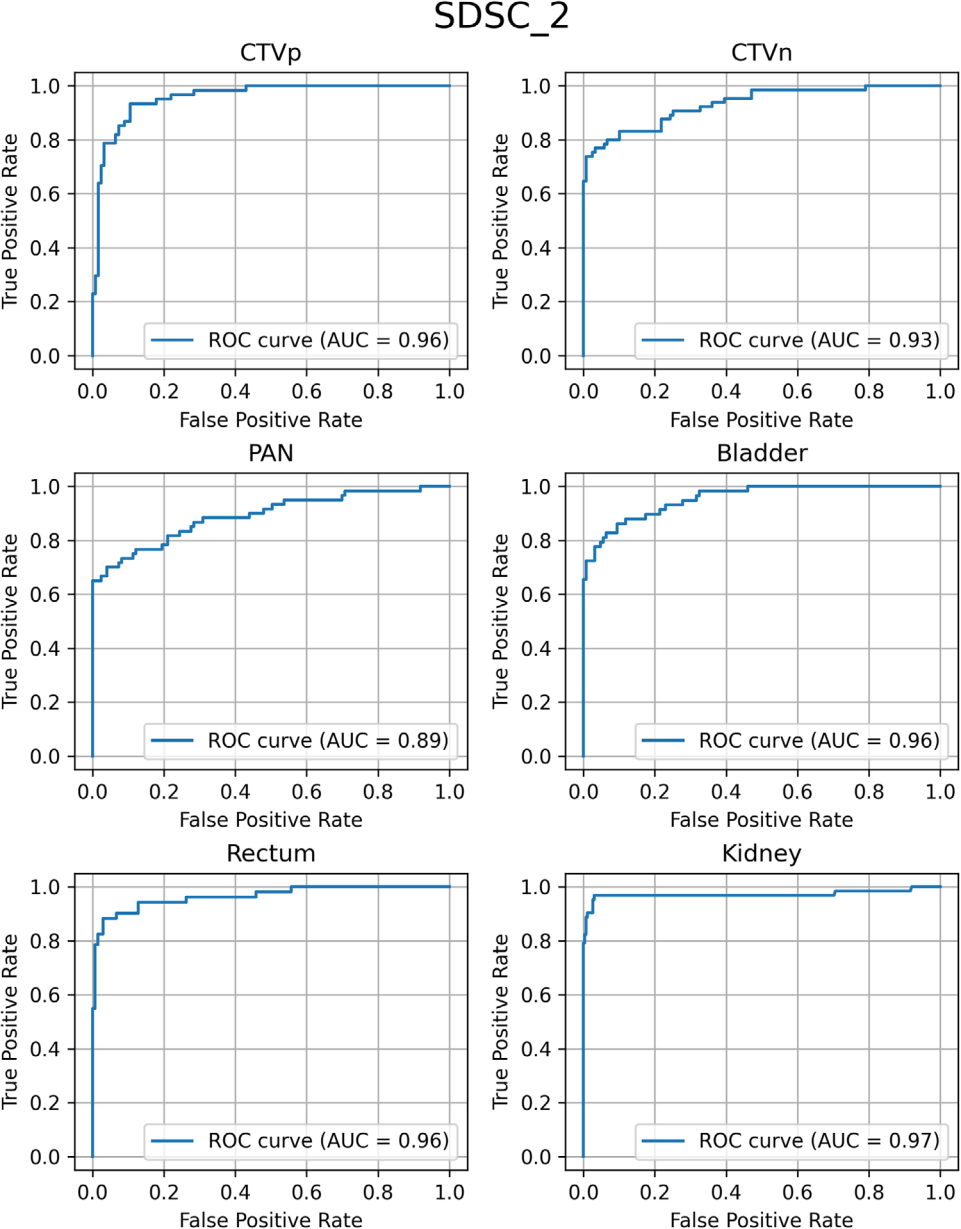
The ROC curves with a surface DSC with a tolerance of 2 mm, the best metric to predict the clinical acceptability of the automatically generated contours. DSC, Dice similarity coefficient

To evaluate the performance more comprehensively, the ROC curves were generated on each metric and each structure, and AUCs were calculated, as shown in Table 4. Again, SDSC_1, SDSC_2, or SDSC_3 was the best metric to predict the clinical acceptability of contours, and HD_100 and HD_95 were not good indicators. The ROC curves for SDSC_2, the best indicator to detect contouring errors based on the AUCs, are presented in Figure 4.

**TABLE 4:** AUCs of each structure and each metric

AUC (95% CI)	UteroCervix	CTVn	PAN	Bladder	Rectum	Kidneys
DSC	0.92 (0.89–0.94)	0.92 (0.89–0.95)	0.86 (0.82–0.89)	0.92 (0.90–0.94)	0.92 (0.89–0.94)	0.97 (0.95–0.99)
HD_100	0.85 (0.81–0.88)	0.75 (0.71–0.79)	0.75 (0.70–0.80)	0.93 (0.90–0.95)	0.81 (0.76–0.84)	0.91 (0.88–0.93)
HD_95	0.87 (0.83–0.89)	0.83 (0.79–0.86)	0.70 (0.65–0.74)	0.96 (0.94–0.97)	0.83 (0.80–0.86)	0.95 (0.92–0.97)
MSD	0.93 (0.91–0.95)	0.92 (0.89–0.94)	0.84 (0.80–0.88)	0.97 (0.96–0.98)	0.92 (0.89–0.94)	0.96 (0.93–0.98)
SDSC 1 mm	0.96 (0.94–0.97)	0.93 (0.90–0.95)	0.90 (0.87–0.93)	0.95 (0.93–0.97)	0.96 (0.94–0.98)	0.95 (0.92–0.97)
SDSC 2 mm	0.96 (0.94–0.97)	0.93 (0.91–0.95)	0.89 (0.86–0.92)	0.96 (0.94–0.97)	0.96 (0.95–0.98)	0.97 (0.95–0.99)
SDSC 3 mm	0.95 (0.93 – 0.96)	0.93 (0.90–0.95)	0.87 (0.83–0.91)	0.97 (0.96–0.98)	0.95 (0.92–0.97)	0.97 (0.95–0.99)
SDSC 4 mm	0.93 (0.91–0.95)	0.92 (0.89–0.94)	0.85 (0.80–0.89)	0.97 (0.95–0.98)	0.93 (0.90–0.96)	0.96 (0.94–0.98)
SDSC 5 mm	0.92 (0.89–0.94)	0.91 (0.88–0.94)	0.83 (0.79–0.88)	0.97 (0.95–0.98)	0.92 (0.88–0.94)	0.95 (0.93–0.97)
SDSC 7 mm	0.90 (0.87–0.93)	0.89 (0.86–0.92)	0.81 (0.76–0.85)	0.96 (0.94–0.97)	0.89 (0.85–0.92)	0.94 (0.92–0.96)
SDSC 10 mm	0.88 (0.85–0.92)	0.85 (0.81–0.88)	0.80 (0.75–0.84)	0.91 (0.88–0.94)	0.85 (0.81–0.89)	0.91 (0.88–0.94)

*Note*: 95% CI for AUCs were derived with the bootstrapping method with *n* = 2000.

Abbreviations: AUC, area under the ROC curve; CI, confidence interval; CTVn, nodal CTV; DSC, Dice similarity coefficient; HD, Hausdorff distance; MSD, mean surface distance; PAN, para‐aortic lymph nodes; SDSC, surface Dice similarity coefficient.

### 3.2 Multi‐metric analysis

We chose combinations of two (DSC_HD) and four (Four_metrics) widely used similarity metrics for contouring studies and the top three and top five most effective surface DSC metrics (Three_SDSC and Five_SDSC) from the single‐metric analysis. Furthermore, the top 5, 7, and 9 most effective metrics from the single metric analysis (Five_metrics, Seven_metrics, and Nine_metrics), and all 11 metrics (All_metrics) were tested in the multi‐metric analysis. Similar to the single‐metric approach, the penalty parameters *C* around 10 gave the best results on average, although there were no substantial differences between the penalty parameters in between 3 and 50. Therefore, we presented all the accuracies here with the penalty parameter *C* = 10.

**FIGURE 5:**
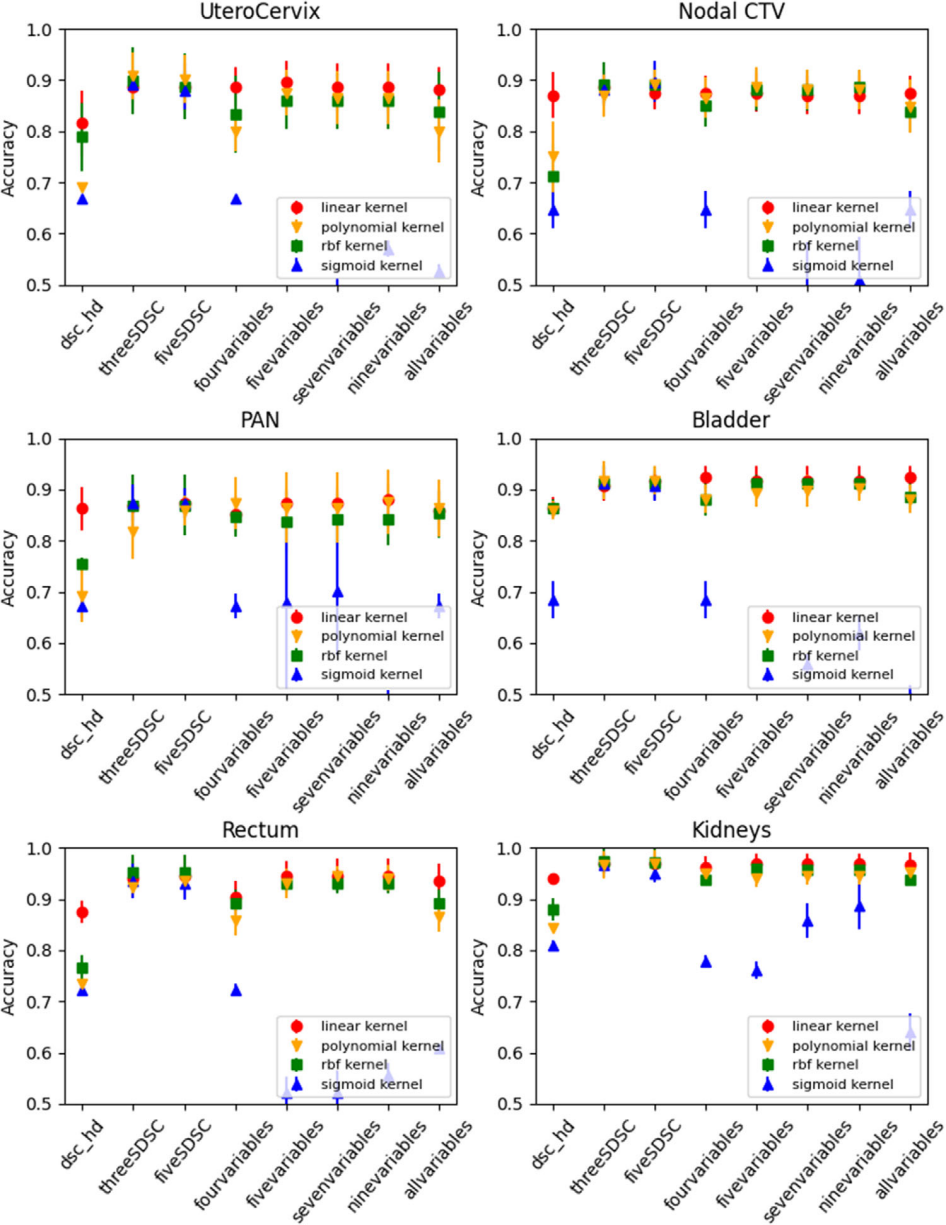
Average accuracies of the SVM model with multiple metrics for each structure. The error bar represents ±1 standard deviation. Four different kernels (linear, polynomial, rbf, and sigmoid) were tested. rbf, radial basis function; SVM, support vector machine

The SVMs with four kernels on different combinations of metrics were trained; the results are shown in Figure 5. We found the optimized values for some kernel parameters (kernel coefficient gamma and degree of the polynomial kernel) from a couple of structures and applied them to the rest of the structures. Most of the kernels had similar performance, but the sigmoid kernel substantially underperformed compared to the other kernels. On average, the model performance with the radial basis function and polynomial kernels fluctuated more with the choice of the metrics than was observed with the linear kernel.

The highest accuracy with the linear kernel was higher than 0.9 for the UteroCervix (0.90 ± 0.02 with Five_metrics), the bladder (0.92 ± 0.02 with Four_metrics), the rectum (0.95 ± 0.03 with Five_metrics), and the kidneys (0.97 ± 0.02 with Five_SDSC) and just below 0.9 for the nodal CTV (0.89 ± 0.04 with Three_SDSC) and the PAN (0.88 ± 0.03 with Nine_metrics). The overall accuracies, sensitivities of detecting erroneous contours, and specificities for the single‐ and multi‐metric analyses with the linear kernel are presented in Tables 5, 6, 7.

**TABLE 5:** Overall accuracies from the single‐metric and multi‐metric analyses, when SVM was used with the linear kernel

Single‐metric	UteroCervix	CTVn	PAN	Bladder	Rectum	Kidneys
DSC	0.82	0.87	0.87	0.88	0.88	0.94
HD_100	0.78	0.72	0.71	0.85	0.75	0.86
HD_95	0.77	0.76	0.66	0.87	0.76	0.91
MSD	0.87	0.88	0.85	0.91	0.89	0.95
SDSC 1 mm	0.91	0.90	0.83	0.89	0.94	0.93
SDSC 2 mm	0.89	0.90	0.86	0.91	0.94	0.97
SDSC 3 mm	0.88	0.88	0.89	0.92	0.92	0.96
SDSC 4 mm	0.89	0.88	0.87	0.91	0.91	0.95
SDSC 5 mm	0.88	0.88	0.87	0.89	0.89	0.94
SDSC 7 mm	0.85	0.87	0.87	0.89	0.86	0.92
SDSC 10 mm	0.80	0.79	0.83	0.86	0.78	0.86

Multi‐metric						
DSC + HD_100	0.82	0.87	0.86	0.86	0.88	0.94
Three_SDSC	0.89	0.89	0.87	0.91	0.94	0.97
Five_SDSC	0.89	0.88	0.87	0.92	0.95	0.97
Four_metrics	0.89	0.88	0.85	0.92	0.90	0.96
Five_metrics	0.90	0.88	0.87	0.92	0.95	0.97
Seven_metrics	0.89	0.87	0.87	0.92	0.95	0.97
Nine_metrics	0.89	0.87	0.88	0.92	0.95	0.97
All_metrics	0.88	0.88	0.86	0.92	0.94	0.97

Abbreviations: CTVn, nodal CTV; DSC, Dice similarity coefficient; HD, Hausdorff distance; MSD, mean surface distance; PAN, para‐aortic lymph nodes; SDSC, surface Dice similarity coefficient; SVM, support vector machine.

**TABLE 6:** Overall sensitivities from the single‐ and multi‐metric analyses, when SVM was used with the linear kernel

Single‐metric	UteroCervix	CTVn	PAN	Bladder	Rectum	Kidneys
DSC	0.59	0.67	0.68	0.69	0.70	0.71
HD_100	0.46	0.33	0.32	0.66	0.22	0.47
HD_95	0.46	0.53	0.00	0.72	0.33	0.65
MSD	0.74	0.73	0.65	0.78	0.78	0.82
SDSC 1 mm	0.82	0.76	0.72	0.76	0.87	0.73
SDSC 2 mm	0.79	0.78	0.68	0.79	0.86	0.90
SDSC 3 mm	0.74	0.74	0.71	0.78	0.81	0.82
SDSC 4 mm	0.77	0.73	0.69	0.76	0.77	0.74
SDSC 5 mm	0.74	0.70	0.67	0.69	0.69	0.71
SDSC 7 mm	0.67	0.64	0.68	0.68	0.59	0.61
SDSC 10 mm	0.53	0.41	0.58	0.57	0.31	0.29

Multi‐metric						
DSC + HD_100	0.61	0.67	0.68	0.69	0.68	0.71
Three_SDSC	0.77	0.76	0.71	0.77	0.87	0.87
Five_SDSC	0.77	0.73	0.69	0.80	0.87	0.89
Four_metrics	0.80	0.73	0.64	0.82	0.77	0.87
Five_metrics	0.82	0.73	0.71	0.80	0.87	0.89
Seven_metrics	0.80	0.73	0.69	0.80	0.87	0.89
Nine_metrics	0.80	0.73	0.69	0.80	0.87	0.89
All_metrics	0.82	0.73	0.66	0.82	0.83	0.87

Abbreviations: CTVn, nodal CTV; DSC, Dice similarity coefficient; HD, Hausdorff distance; MSD, mean surface distance; PAN, para‐aortic lymph nodes; SDSC, surface Dice similarity coefficient; SVM, support vector machine.

**TABLE 7:** Overall specificities from the single‐ and multi‐metric analyses, when SVM was used with the linear kernel

Single‐metric	UteroCervix	CTVn	PAN	Bladder	Rectum	Kidneys
DSC	0.94	0.98	0.97	0.96	0.94	1.00
HD_100	0.94	0.93	0.90	0.93	0.96	0.95
HD_95	0.92	0.89	0.93	0.94	0.93	0.97
MSD	0.93	0.96	0.95	0.98	0.93	0.98
SDSC 1 mm	0.95	0.97	0.89	0.95	0.97	0.97
SDSC 2 mm	0.94	0.97	0.95	0.97	0.97	0.99
SDSC 3 mm	0.94	0.96	0.98	0.99	0.97	0.99
SDSC 4 mm	0.94	0.96	0.97	0.99	0.97	1.00
SDSC 5 mm	0.94	0.98	0.98	0.99	0.97	1.00
SDSC 7 mm	0.94	0.99	0.98	0.99	0.96	1.00
SDSC 10 mm	0.94	1.00	0.96	1.00	0.96	1.00

Multi‐metric						
DSC + HD_100	0.92	0.98	0.96	0.94	0.95	1.00
Three_SDSC	0.94	0.96	0.95	0.98	0.97	0.99
Five_SDSC	0.94	0.96	0.97	0.98	0.98	0.99
Four_metrics	0.93	0.96	0.96	0.98	0.96	0.98
Five_metrics	0.94	0.96	0.96	0.98	0.98	0.99
Seven_metrics	0.93	0.95	0.97	0.98	0.98	0.99
Nine_metrics	0.93	0.95	0.98	0.98	0.98	0.99
All_metrics	0.91	0.96	0.96	0.98	0.98	0.99

Abbreviations: CTVn, nodal CTV; DSC, Dice similarity coefficient; HD, Hausdorff distance; MSD, mean surface distance; PAN, para‐aortic lymph nodes; SDSC, surface Dice similarity coefficient; SVM, support vector machine.

## 4 DISCUSSION

In this study, we demonstrated that errors in a contour can be detected by being compared with another independently generated contour. By choosing appropriate similarity metrics and using the SVM classification algorithm, we were able to achieve an accuracy higher than 0.9 for most of the structures. Furthermore, we reported the optimized thresholds from our dataset in Table 2 so that anyone can use these thresholds for their QA system for the pelvic structures.

From this study, we showed that the surface DSC with a tolerance of 1–3 mm is the best similarity metric to detect contouring errors. Using combinations of multiple metrics did not improve the accuracy of detecting contouring errors. This could be because we did not have enough data to fine‐tune the thresholds to substantially improve the results of the single‐metric approach. In addition, because most of the metrics are already strongly correlated with each other, the classification model might have not been able to learn useful information from the additional metrics. In any case, using a single metric to flag incorrect contours performed as accurate as or even more accurate than did combinations of multiple metrics and makes it easier for users to interpret the results. Furthermore, considering the variations in the sizes and shapes of the structures used in this study, the single‐metric approach should be feasible for most of the structures in various treatment sites. Therefore, we believe that the single‐metric approach, especially using the surface DSC metric, is the best approach to detect contouring errors utilizing two autocontouring systems and is expandable to other treatment sites. Although preliminary, this work indicates that an SDSC_2 threshold of 0.54 may be a reasonable starting point for a wide variety of structures.

In the single‐metric analysis, we found that the volumetric DSC and MSD are effective indicators for determining the similarity between the two contours in terms of error detection. On the other hand, the HDs (HD_100 and HD_95) often failed to detect contouring errors in our QA method, demonstrating that most realistic contouring errors were not caused by a substantial failure in a single or small part of the contour and possibly explaining why the surface DSC was very effective at detecting contouring errors. As the surface DSC only compares the volume of the shell, the metric may indicate the overall similarity between the two contours near the surface. MSD is similar, but any small discrepancy between the two contours in each calculation point can contribute to the MSD. On the other hand, the surface DSC is more effective as the user can choose the tolerance value and anything below the tolerance will not contribute to reducing the surface DSC. For the surface DSC with a tolerance higher than 5 mm, however, the accuracy decreased substantially; thus, it is recommended to use the surface DSC with a tolerance of less than 5 mm to compare two contours in future studies.

**FIGURE 6:**
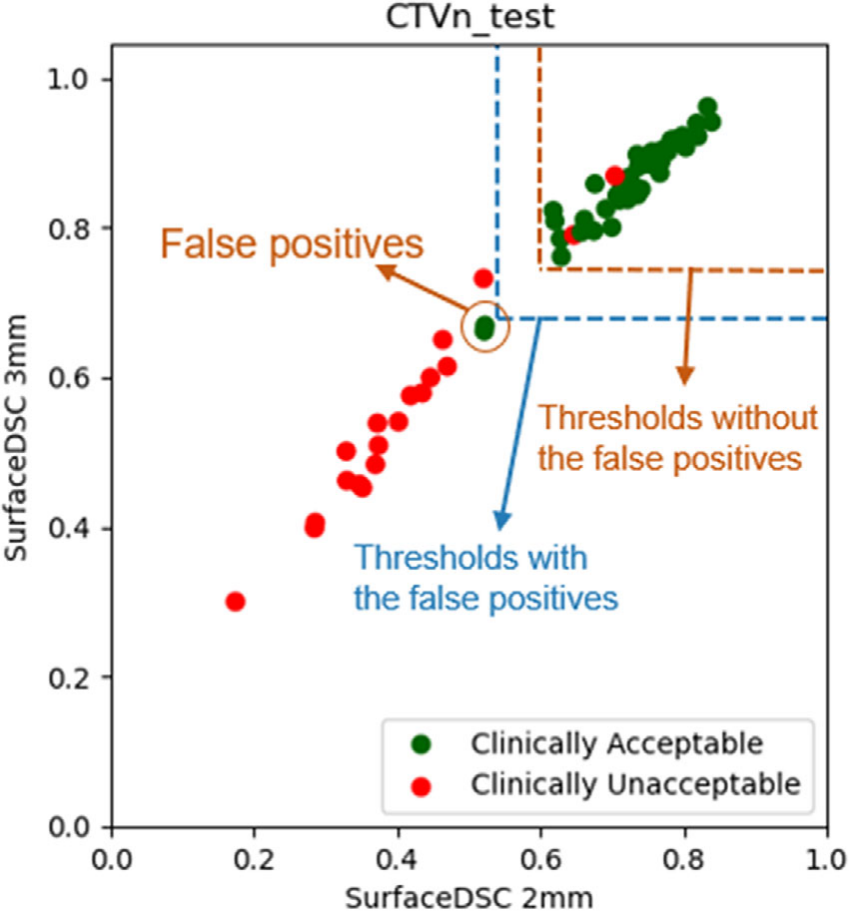
False positives can make the thresholds more generous (blue dashed lines) than the desired thresholds (brown dashed lines) and result in having more false negatives in clinical situations

One of the weaknesses of this study is that the 49 internal CT scans were from the training dataset of the verification autocontouring system, although 38 external CT scans were independent from it. This makes the most of the verification autocontours to be accurately predicted on the internal CT scan and, therefore, substantially reduces the false positives (clinically acceptable reference contours classified as clinically unacceptable). From our preliminary study, the false positives mostly occurred when the verification contours were unacceptable, whereas the reference contours were acceptable. This reduction in false positives probably overestimated the performance of the QA method with the given autocontouring systems. Yet, a high false‐positive rate misleads the SVM algorithm determining the accurate thresholds. The algorithm is incorrect for false positives as it does not correctly predict the status of the reference contour. However, the algorithm could have been correct with the same exact pair of contours with the reference and the verification contours swapped. Having a high false‐positive rate would make the thresholds to be more generous, as shown in Figure 6, and results in increasing false negatives (clinically unacceptable reference contours classified as clinically acceptable), the least desired situations in any automatic contouring QA methods. This is an inherent limitation of an automatic contouring QA method using scalar quantities, and we intentionally decreased false‐positive rates and overestimated the overall accuracy to overcome the limitation. Fortunately, having a high false‐positive rate is not critical in a clinical scenario. We will provide both reference and verification contours to the users when the discrepancy between the two contours is substantial, and the user will select the better contour. If both contours were provided for the false‐positive case, the user can quickly review the reference contour and use it as is. Therefore, we believe that deriving the more accurate thresholds for the contouring QA method outweighs calculating the more accurate performance that is specific to our autocontouring systems datasets. Moreover, as we currently implement and test this system clinically, we will reassess the performance of the QA system with independent data in the future.

**FIGURE 7:**
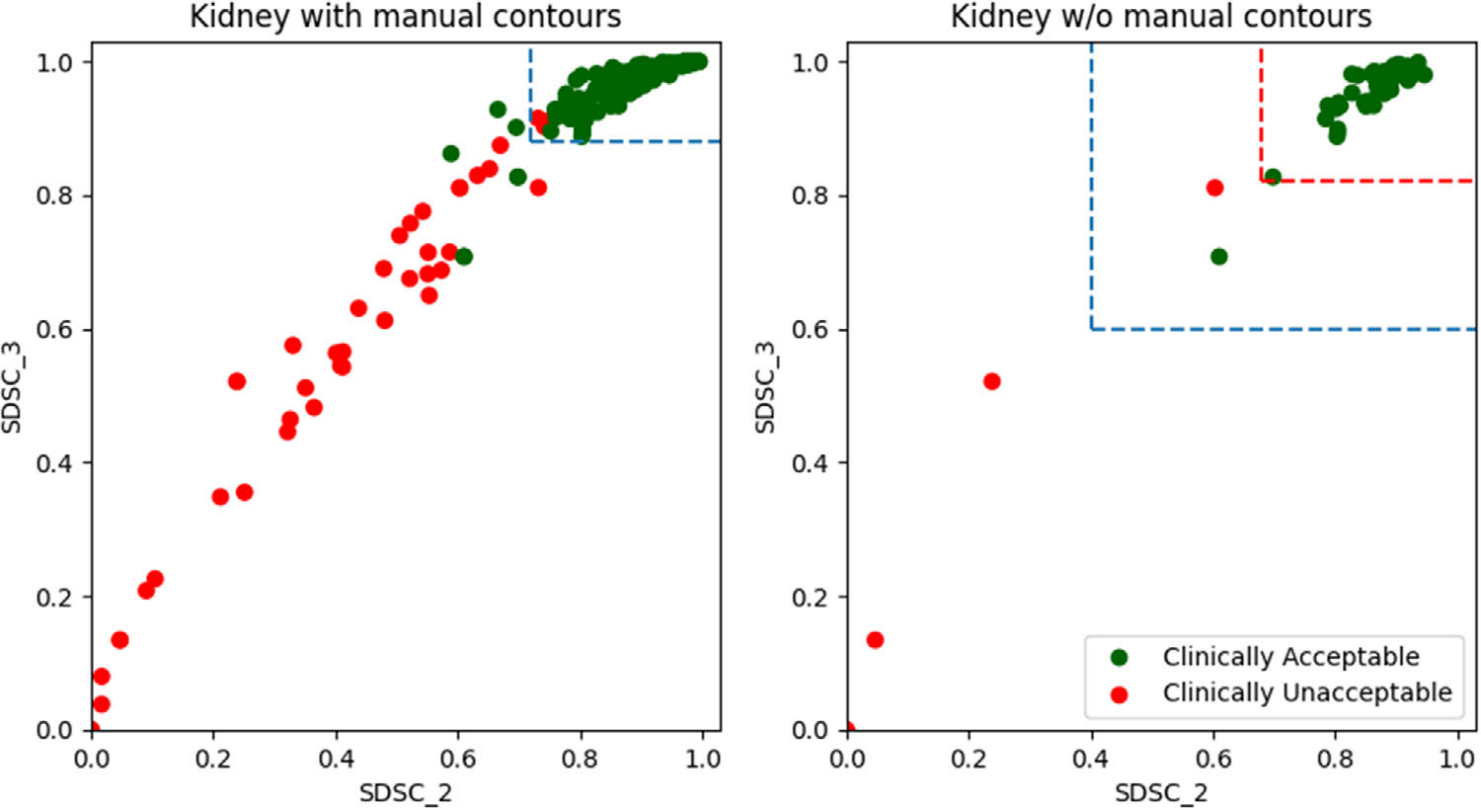
The surface DSC distributions of the clinically acceptable and unacceptable kidney contours with (left) and without (right) the manually generated contours. The thresholds can be confidently determined with the manual contours, whereas the threshold can be anywhere between the blue and red dashed lines without the manual contours due to insufficient amount of data. DSC, Dice similarity coefficient

Another concern in this study was that the majority of the unacceptable contours were manually introduced to mimic a potential error that can be made by a human or a deep learning algorithm. Consequently, the distributions of the metrics and the derived accuracies in this study might not fully reflect the actual performance of the contour QA model on the autocontouring systems. However, in our preliminary study, the metric points corresponding to both the clinically acceptable and unacceptable contours near the thresholds were not sufficient, as shown in Figure 7. The thresholds could have been chosen anywhere in between the red and blue dashed lines on the right part of Figure 7, when the manual contours were not included. Therefore, predicting the verification autocontours on its training dataset and adding the manual contours helped us determine more robust metrics for the contour QA models.

As our automatic contouring QA method is not perfect, it is still mandatory for experts to review all the contours. However, an automatic contouring QA method can provide a second opinion on both manually and automatically generated contours and potentially detect the errors that experts might miss. Furthermore, the performance of the contouring QA method is improved as the performance of the verification autocontouring system is improved. With the rapid advancement of deep learning architectures for semantic segmentation tasks, we are observing great progress in autocontouring systems, and they will eventually achieve human‐level performance in the near future. At that point, the contouring QA method will detect almost all the contouring errors except for the subtle failures. Moreover, Cao et al.^19^ showed that suboptimal contours from an autocontouring system do not lead to systematic differences in dosimetric plan quality compared to that with the manually generated contours. This tells us that if we can avoid any substantial contouring errors, the final plan would not be significantly different from the plan generated with flawless contours. With all that, a proper use of our automatic contouring QA method can contribute to the safety of radiation treatment.

## 5 CONCLUSIONS

We demonstrated that the discrepancy between two independently generated contours is a strong indicator of an error in one of the contours. The most accurate similarity metric to detect contouring errors was surface DSC with a tolerance of 1, 2, or 3 mm. With this approach, we were able to achieve the error detection accuracy higher than 0.9 for most of the targets and critical structures in the female pelvis. The contouring QA method can be used to automatically detect errors in autocontours to reduce the risks associated with the use of automated radiotherapy tools. We will validate this QA method in other sites and structures in the future.

## CONFLICT OF INTEREST

This work was partially funded by the National Cancer Institute and Varian Medical Systems.

## AUTHOR CONTRIBUTION

The authors confirm contribution to the paper as follows: study conception and design: Dong Joo Rhee, Carlos E. Cardenas, Surendra Prajapati, Stephen F. Kry, Kristy K. Brock, Beth M. Beadle, Laurence E. Court; deep learning model development: Dong Joo Rhee, Bastien Rigaud, Lifei Zhang, Kristy Brock; data generation: Chidinma P. Anakwenze Akinfenwa, Anuja Jhingran, William Shaw, Frederika O'Reilly, Jeannette Parkes, Hester Burger, Nazia Fakie, Chris Trauernicht, Hannah Simonds; analysis and interpretation of results: Dong Joo Rhee, Carlos E. Cardenas, Laurence E. Court; draft manuscript preparation: Dong Joo Rhee, Laurence E. Court. All authors reviewed the results and approved the final version of the manuscript.
